# Low coverage whole genome sequencing enables accurate assessment of common variants and calculation of genome-wide polygenic scores

**DOI:** 10.1186/s13073-019-0682-2

**Published:** 2019-11-26

**Authors:** Julian R. Homburger, Cynthia L. Neben, Gilad Mishne, Alicia Y. Zhou, Sekar Kathiresan, Amit V. Khera

**Affiliations:** 1 Color Genomics, 831 Mitten Road, Suite 100, Burlingame, CA 94010 USA; 2 Verve Therapeutics, Cambridge, MA USA; 3 Center for Genomic Medicine and Cardiology Division, Department of Medicine, Massachusetts General Hospital, Simches Research Building | CPZN 6.256, Boston, MA 02114 USA; 4 Cardiovascular Disease Initiative, Broad Institute of MIT and Harvard, Cambridge, MA 02142 USA; 5 Harvard Medical School, Boston, MA 02115 USA

**Keywords:** Genome-wide polygenic score, Low coverage whole genome sequencing, Coronary artery disease, Breast cancer, Atrial fibrillation

## Abstract

**Background:**

Inherited susceptibility to common, complex diseases may be caused by rare, pathogenic variants (“monogenic”) or by the cumulative effect of numerous common variants (“polygenic”). Comprehensive genome interpretation should enable assessment for both monogenic and polygenic components of inherited risk. The traditional approach requires two distinct genetic testing technologies—high coverage sequencing of known genes to detect monogenic variants and a genome-wide genotyping array followed by imputation to calculate genome-wide polygenic scores (GPSs). We assessed the feasibility and accuracy of using low coverage whole genome sequencing (lcWGS) as an alternative to genotyping arrays to calculate GPSs.

**Methods:**

First, we performed downsampling and imputation of WGS data from ten individuals to assess concordance with known genotypes. Second, we assessed the correlation between GPSs for 3 common diseases—coronary artery disease (CAD), breast cancer (BC), and atrial fibrillation (AF)—calculated using lcWGS and genotyping array in 184 samples. Third, we assessed concordance of lcWGS-based genotype calls and GPS calculation in 120 individuals with known genotypes, selected to reflect diverse ancestral backgrounds. Fourth, we assessed the relationship between GPSs calculated using lcWGS and disease phenotypes in a cohort of 11,502 individuals of European ancestry.

**Results:**

We found imputation accuracy *r*^2^ values of greater than 0.90 for all ten samples—including those of African and Ashkenazi Jewish ancestry—with lcWGS data at 0.5×. GPSs calculated using lcWGS and genotyping array followed by imputation in 184 individuals were highly correlated for each of the 3 common diseases (*r*^2^ = 0.93–0.97) with similar score distributions. Using lcWGS data from 120 individuals of diverse ancestral backgrounds, we found similar results with respect to imputation accuracy and GPS correlations. Finally, we calculated GPSs for CAD, BC, and AF using lcWGS in 11,502 individuals of European ancestry, confirming odds ratios per standard deviation increment ranging 1.28 to 1.59, consistent with previous studies.

**Conclusions:**

lcWGS is an alternative technology to genotyping arrays for common genetic variant assessment and GPS calculation. lcWGS provides comparable imputation accuracy while also overcoming the ascertainment bias inherent to variant selection in genotyping array design.

## Background

Cardiovascular disease and cancer are common, complex diseases that remain leading causes of global mortality [1]. Long recognized to be heritable, recent advances in human genetics have led to consideration of DNA-based risk stratification to guide prevention or screening strategies. In some cases, such conditions can be caused by rare, “monogenic” pathogenic variants that lead to a several-fold increased risk—important examples are pathogenic variants in *LDLR* that cause familial hypercholesterolemia and pathogenic variants in *BRCA1* and *BRCA2* that underlie hereditary breast and ovarian cancer syndrome. However, the majority of individuals afflicted with these diseases do not harbor any such pathogenic variants. Rather, the inherited susceptibility of many complex traits and diseases is often “polygenic,” driven by the cumulative effect of numerous common variants scattered across the genome [2].

Genome-wide polygenic scores (GPSs) provide a way to integrate information from numerous sites of common variation into a single metric of inherited susceptibility and are now able to identify individuals with a several-fold increased risk of common, complex diseases, including coronary artery disease (CAD), breast cancer (BC), and atrial fibrillation (AF) [3]. For example, for CAD, we previously noted that 8% of the population inherits more than triple the normal risk on the basis of polygenic variation, a prevalence more than 20-fold higher than monogenic familial hypercholesterolemia variants in *LDLR* that confer similar risk [3].

Comprehensive genome interpretation for common, complex disease therefore could involve both high-fidelity sequencing of important driver genes to identify rare monogenic risk variants and a survey of all common variants across the genome to enable GPS calculation. High coverage whole genome sequencing (hcWGS; for example, 30× coverage) will likely emerge as a single genetic testing strategy, but current prices remain a barrier to large-scale adoption. Instead, the traditional approach has mandated use of two distinct genetic testing technologies—high coverage next generation sequencing (NGS) of important genes to detect pathogenic variants and a genome-wide genotyping array followed by imputation to calculate GPSs.

Low coverage whole genome sequencing (lcWGS; for example, 0.5× coverage) followed by imputation is a potential alternative to genotyping arrays for assessing the common genetic variants needed for GPS calculations. Several recent studies have demonstrated the efficiency and accuracy of lcWGS for other applications of statistical genetics, including local ancestry deconvolution, and complex trait association studies [4–7].

We developed a pipeline for common genetic variant imputation using lcWGS data on samples from the 1000 Genomes Project (1KGP) [8] and Genome in a Bottle (GIAB) Consortium [9] and herein demonstrate imputation accuracy for lcWGS similar to genotyping arrays. Using three recently published GPSs for CAD [3], BC [10], and AF [3], we show high technical concordance in GPSs calculated from lcWGS and genotyping arrays. Finally, using our pipeline in a large European population seeking genetic testing, we observe similar GPS risk stratification performance as previously published array-based results [3, 10].

## Methods

### Study design

**Fig. 1:**
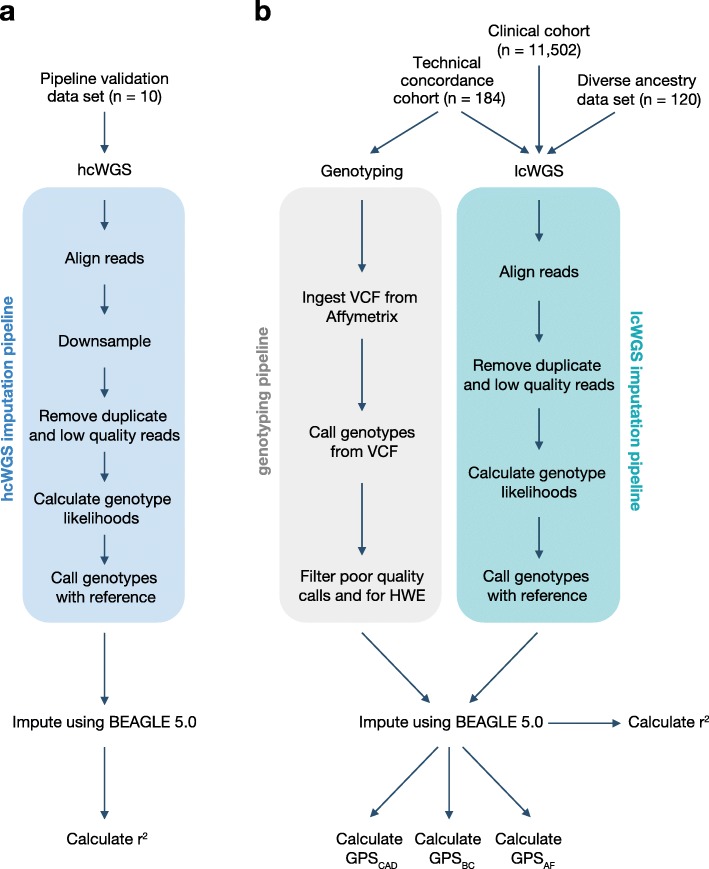
Study design and imputation pipelines. The study design has four groups: **a** pipeline validation data set and **b** technical concordance cohort, diverse ancestry data set, and clinical cohort. The imputation pipeline for each group is depicted. hcWGS, high coverage whole genome sequencing; lcWGS, low coverage whole genome sequencing; HWE, Hardy-Weinberg equilibrium; GPS, genome-wide polygenic score; CAD, coronary artery disease; BC, breast cancer; AF, atrial fibrillation

The study design is summarized in Fig. 1 and described in detail below. The pipeline validation data set (*n* = 10) was used to assess imputation accuracy for common genetic variants (Fig. 1a). The technical concordance cohort (*n* = 184) was used to assess the correlation between three previously published GPSs for CAD [3], BC [10], and AF [3] from lcWGS and genotyping arrays (Fig. 1b). The diverse ancestry data set (*n* = 120) was used to assess imputation accuracy for common genetic variants and performance of GPS_CAD_, GPS_BC_, and GPS_AF_ (Fig. 1b). The clinical cohort (*n* = 11,502) was used to assess performance of GPS_CAD_, GPS_BC_, and GPS_AF_ in a large European population seeking genetic testing (Fig. 1b).

### Data set and cohort selection

The pipeline validation data set included seven globally representative samples from 1KGP populations (HG02155, NA12878, HG00663, HG01485, NA21144, NA20510, and NA19420; Additional file 1: Table S1) [8] and a trio of Ashkenazi samples (NA24385, NA24143, and NA24149) from the GIAB Consortium (Fig. 1a) [9].

The technical concordance cohort included DNA samples from 184 individuals whose healthcare provider had ordered a Color multi-gene panel test (Fig. 1b). All individuals (1) had 85% or greater European genetic ancestry calculated using fastNGSadmix [11] using 1KPG as the reference panel, (2) self-identified as “Caucasian,” and (3) did not have pathogenic or likely pathogenic variants in the multi-gene NGS panel test, as previously described [12] (Additional file 2: Supplementary Methods). Demographics are provided in Additional file 1: Table S2. All phenotypic information was self-reported by the individual through an online, interactive health history tool. Of the 184 individuals, 61 individuals reported having a personal history of CAD (defined here as a myocardial infarction or coronary artery bypass surgery), 62 individuals reported no personal history of CAD, and 61 individuals reported no personal history of CAD but were suspected to have a high GPS_CAD_ based on preliminary analysis. This preliminary analysis included imputation from multi-gene panel and off-target sequencing data, which has been shown to have similar association statistics and effect sizes compared to genotyping arrays [4]. These individuals were included in the technical concordance cohort to artificially create a relatively uniform distribution of GPS_CAD_ in the data set. Correlation coefficients between GPS_CAD_ from lcWGS and genotyping array were calculated after removing the 61 individuals who were suspected to have a high GPS_CAD_ based on multi-gene panel and off-target sequencing data to avoid artificial inflation of the correlation coefficient. Two individuals who reported no personal history of CAD but were suspected to have a high GPS_CAD_ failed genotyping (quality control call rate of < 97%) and lcWGS (overall coverage of < 0.5×), leaving a total of 182 individuals for analyses.

The diverse ancestry data set included a total of 120 samples from the following populations from 1KGP: Han Chinese in Beijing, China (CHB); Yoruba in Ibadan, Nigeria (YRI); Gujarati Indian from Houston, Texas (GIH); Americans of African Ancestry in Southwest USA (ASW); Mexican Ancestry from Los Angeles, USA (MXL); and Puerto Ricans from Puerto Rico (PUR) (Additional file 1: Table S3 and Additional file 3: Figure S1) [8]. Four samples, including NA18917 and NA19147 from the YRI population and NA19729 and NA19785 from the MXL population, were below the target 0.5× coverage and removed from analyses.

The clinical cohort included DNA samples from 11,502 individuals whose healthcare provider had ordered a Color multi-gene panel test (Fig. 1b). All individuals (1) had 90% or greater European genetic ancestry calculated using fastNGSadmix [11] using 1KPG as the reference panel; (2) self-identified as “Caucasian”; (3) provided history of whether they had a clinical diagnosis of CAD, BC, or AF, via an online, interactive health history tool; and (4) did not have pathogenic or likely pathogenic variants detected in the multi-gene sequencing panel test, as previously described [12] (Additional file 2: Supplementary Methods). Demographics are provided in Additional file 1: Table S2.

### Whole genome sequencing

DNA was extracted from blood or saliva samples and purified using the Perkin Elmer Chemagic DNA Extraction Kit (Perkin Elmer, Waltham, MA) automated on the Hamilton STAR (Hamilton, Reno, NV) and the Chemagic Liquid Handler (Perkin Elmer, Waltham, MA). The quality and quantity of the extracted DNA were assessed by UV spectroscopy (BioTek, Winooski, VT). High molecular weight genomic DNA was enzymatically fragmented and prepared using the Kapa HyperPlus Library Preparation Kit (Roche Sequencing, Pleasanton, CA) automated on the Hamilton Star liquid handler and uniquely tagged with 10 bp dual-unique barcodes (IDT, Coralville, IA). Libraries were pooled together and loaded onto the NovaSeq 6000 (Illumina, San Diego, CA) for 2 × 150 bp sequencing.

For the pipeline validation data set, all samples underwent WGS with mean coverage of 13.22× (range 7.82× to 17.30×); downsampling was then performed using SAMtools [13] to simulate lcWGS. For the technical concordance cohort, all samples underwent lcWGS with mean coverage of 1.24× (range 0.54× to 1.76×). Imputed genotypes were compared with published, high-confidence known genotypes from 1KGP [8] and the GIAB Consortium [9]. For the diverse ancestry data set, all samples underwent lcWGS with mean coverage of 0.89× (range 0.68× to 1.24×). For the clinical cohort, all samples underwent lcWGS with mean coverage of 0.95× (range 0.51× to 2.57×).

### Downsampling

For the pipeline validation data set, aligned reads were downsampled using SAMtools [13] to 2.0×, 1.0×, 0.75×, 0.5×, 0.4×, 0.25×, and 0.1× coverage. For the technical concordance cohort, aligned reads were downsampled to 1.0×, 0.75×, 0.5×, 0.4×, 0.25×, and 0.1× coverage. In a few cases in the technical concordance cohort, the primary samples had fewer reads than the target downsample. In those situations, all of the reads were retained. For example, if the primary sample only had 0.8× coverage, when downsampled to 1.0×, all reads were retained. Downsampling was repeated using two independent seeds in SAMtools. Once the downsampled data was generated, the imputation was repeated to generate imputed genotypes using only the downsampled reads.

### Imputation site selection

All data sets and cohorts were imputed to a set of autosomal SNP and insertion-deletion (indel) sites from 1KGP with greater than 1% allele frequency in any of the five 1KGP super populations (African, American, East Asian, European, and South Asian) [8], for a total of 21,770,397 sites. This is hereafter referred to as the “imputation SNP loci.” Multi-allelic SNPs and indels were represented as two biallelic markers for imputation.

### Genotype likelihood calculations and imputation

Genotype likelihood calculations and imputation were performed independently for each sample. Sequence reads were aligned with the human genome reference GRCh37.p12 using the Burrows-Wheeler Aligner (BWA) [14], and duplicate and low quality reads were removed. Genotype likelihoods were then calculated at each of the biallelic SNP loci in the imputation SNP loci that were covered by one or more sequencing reads called using the mpileup command implemented in bcftools version 1.8 [15]. Indels or multi-allelic sites were not included in this first genotype likelihood calculation. Reads with a minimum mapping alignment quality of 10 or greater and bases with a minimum base quality of 10 or greater were included. Genotype likelihoods at each observed site were then calculated using the bcftools call command with allele information corresponding to the imputation SNP loci. This procedure discarded calls with indels or calls where the observed base did not match either the reference or the expected alternate allele for the SNP locus.

To convert genotype likelihoods into genotype calls at all imputation SNP loci, two distinct calculations were performed. First, genotypes at imputation SNP loci covered by at least one read were inferred. Genotype calling was performed using the genotype likelihood option implemented in BEAGLE 4.1 [14]. This step is a reference-aware genotype calling step and produces posterior probabilities of genotypes only at sites with at least one read. This algorithm is implemented only in BEAGLE 4.1 [16]. This inference used default BEAGLE 4.1 [16] parameters except with a model scale parameter of 2 and the number of phasing iterations to 0. A custom reference panel was constructed for each sample being imputed by selecting the 250 most similar samples to that sample from 1KGP Phase 3 [8] release using Identity-by-State (IBS) comparison. A reference panel size of 250 was selected to best balance imputation run time and accuracy (Additional file 3: Figure S1). To ensure that IBS values were comparable across samples, a set of regions consistently sequenced at high depth (> 20×) across all samples was utilized. Inclusion of related samples in an imputation reference panel can artificially increase imputation accuracy; therefore, when imputation was performed on samples included in 1KGP Phase 3 release, that sample and any first and second degree related samples (as inferred by the 1KGP data release using genetic data) were excluded from the custom reference panel.

To generate genotypes at all of the remaining untyped sites, a second round of imputation was performed using BEAGLE 5.0 [16]. This imputation used default settings and included the full 1KGP as the imputation reference panel [8]. To note, when performing analysis using 1KGP samples [8], any related individuals were removed. Each sample then had imputed genotype calls at each of the imputation SNP loci. Indels and multi-allelic sites were included in this second genotype likelihood calculation.

### Genotyping array

DNA was extracted from blood or saliva samples and purified using the Perkin Elmer Chemagic DNA Extraction Kit (Perkin Elmer, Waltham, MA) automated on the Hamilton STAR (Hamilton, Reno, NV) and the Chemagic Liquid Handler (Perkin Elmer, Waltham, MA). The quality and quantity of the extracted DNA were assessed by UV spectroscopy (BioTek, Winooski, VT).

DNA was genotyped on the Axiom UK Biobank Array by Affymetrix (Santa Clara, CA). Genotypes were filtered according to the manufacturer’s recommendations, removing loci with greater than 5% global missingness and those that significantly deviated from the Hardy-Weinberg equilibrium. In addition, all A/T and G/C SNPs were removed due to potential strand inconsistencies. After applying the above quality filtering and filtering for ambiguous SNP sites, 748,187 SNPs out of an original 830,115 polymorphic sites remained. Each of the remaining SNP orientation was aligned with the hg19 reference sequence to correctly code the reference alleles as allele 1, matching the sequencing data.

To generate genotypes at all of the remaining untyped sites, imputation was performed using BEAGLE 5.0 [16]. This imputation used default settings and included the full 1KGP as the imputation reference panel [8]. To note, when performing analysis using 1KGP samples, any related individuals were removed. Each sample then had imputed genotype calls at each of the imputation SNP loci.

### Imputation accuracy and quality assessment

Imputation accuracy for 1KGP and GIAB samples was calculated by comparing imputation results with previously released genotypes, excluding regions marked as low confidence by GIAB.

Imputation accuracy on the genotyped samples was assessed on 470,363 sites that were included in the genotyping array and in the imputation SNP loci at different allele frequency buckets: 257,362 sites with greater than 5% allele frequency, 119,978 sites between 1 and 5% allele frequency, and 93,022 sites with less than 1% allele frequency. Imputation quality was assessed through site-specific dosage *r*^2^ comparing with genotype values from the genotyping array.

### GPS selection

The GPSs for CAD [3], BC [10], and AF [3] were previously published and selected based on their demonstrated ability to accurately predict and stratify disease risk as well as identify individuals at risk comparable to monogenic disease. GPS_CAD_ contained 6,630,150 polymorphisms, GPS_BC_ contained 3820 polymorphisms, and GPS_AF_ contained 6,730,541 polymorphisms. All loci included in these scores were included in the imputation SNP loci.

### GPS normalization

In the clinical cohort, raw GPSs were normalized by taking the standardized residual of the predicted score after correction for the first 10 principal components (PCs) of ancestry [17]. PCs were calculated by projecting lcWGS samples into 10 dimensional PC analyses (PCAs) space using the LASER program [18]. A combination of samples from 1KGP [8] and the Human Origins [19] project were used as a reference for the projection.

## Results

### Development and validation of imputation pipeline for lcWGS

Previous studies have evaluated the potential use of lcWGS in local ancestry deconvolution, complex trait association studies, and detection of rare genetic variants [4–6]. To assess the feasibility and accuracy of this approach for GPSs, we first developed an imputation pipeline that reads raw fastq sequence data and generates a vcf with imputed site information at 21.7 million sites (imputation SNP loci) (Fig. 1a, b). Briefly, reads are aligned to the reference genome and filtered for duplicates and low quality. Using this BAM file, we then calculate genotype likelihoods and impute expected genotypes using 1KGP as the imputation reference panel.

**Fig. 2:**
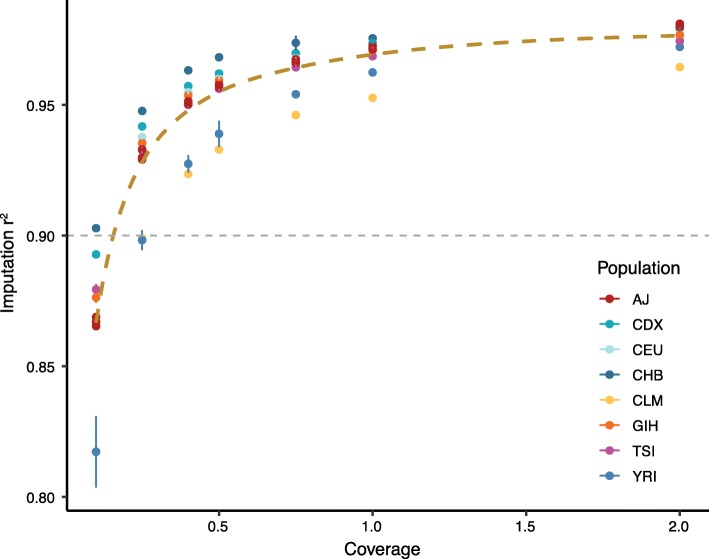
Assessment of imputation performance in the pipeline validation data set. Downsampling from 30× to 0.1× showed that lcWGS accuracy was above 0.90 *r*^2^ for all samples at 0.5× (*n* = 4 independent random seeds for each sample and coverage value; error bars are 95% confidence intervals). The thick brown dashed line is a smoothed trendline of the average imputation quality while the thin gray dashed line demonstrates previously reported imputation quality from a genotyping array (*r*^2^ = 0.90) [4]. AJ, Ashkenazi Jewish; CDX, Chinese Dai in Xishuangbanna, China; CEU, Utah residents with Northern and Western European ancestry; CHB, Han Chinese in Beijing, China; CLM, Colombians from Medellin, Colombia; GIH, Gujarati Indian from Houston, Texas; TSI, Toscani in Italia; YRI, Yoruba in Ibadan, Nigeria

To validate this imputation pipeline, we performed hcWGS and downsampling on seven samples from different 1KGP populations and a trio of Ashkenazi Jewish GIAB samples (pipeline validation data set) to varying depths of coverage from 2.0× to 0.1× (Additional file 1: Table S1) [9]. We used the published genotype calls for each of these samples at all 21 million imputation SNP loci as truth data and found that imputation accuracy was above 0.90 *r*^2^ for all samples at 0.5× and higher (Fig. 2). As expected, this was correlated with sequencing depth, with diminishing gains observed at coverages above 1.0×. While imputation accuracy was similar across diverse populations, it was slightly reduced in the Colombian sample (HG01485), likely due to complex local ancestry related to admixture, and in the Yoruban sample (NA19240), likely due to the shorter blocks of linkage disequilibrium and higher genetic diversity in Africa [8]. Taken together, these data suggest that at sequencing depth at or above 0.5×, our pipeline has similar imputation accuracy to genotyping array-based imputation across individuals from multiple populations. As such, we set 0.5× as a quality control for success and removed samples with coverage below this threshold in subsequent analyses.

### Technical concordance between GPSs calculated from lcWGS and genotyping array

To assess the technical concordance of using lcWGS to calculate GPSs, we performed low coverage sequencing and used genotyping arrays on DNA from 184 individuals (technical concordance cohort) (Fig. 1b). This concordance assessment was restricted to individuals of European ancestry to most closely align with the populations used for GPS training and validation.

We first compared the lcWGS genotype dosages with a subset of variants directly genotyped (*n* = 470,362) on the genotyping array to assess imputation performance. Assuming the typed loci called on the genotyping array as “true,” we observed an average imputation *r*^2^ > 0.90 at 0.5× depth for variants with global minor allele frequency (MAF) greater than 5% and for variants with European MAF greater than 5% (Additional file 3: Figure S2). As expected, imputation accuracy was highest for variants with higher MAF. For lower frequency variants, we saw a reduction in imputation accuracy, as expected, with *r*^2^ > 0.85 for variants at 1 to 5% MAF and *r*^2^ > 0.80 for variants less than 1% global MAF. Taken together, this demonstrates that lcWGS has high accuracy in this test setting.

**Fig. 3:**
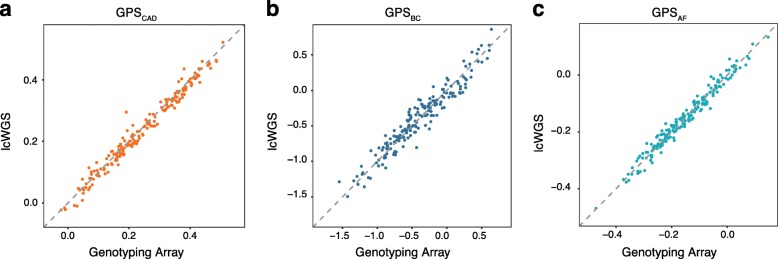
Correlation of GPSs between genotyping array and lcWGS in the technical concordance cohort. **a** GPS_CAD_ calculated using lcWGS was highly correlated (*r*^2^ = 0.98) with those calculated using genotyping array (*n* = 182). **b** GPS_BC_ calculated using lcWGS was highly correlated (*r*^2^ = 0.93) with those calculated using genotyping array (*n* = 182). **c** GPS_AF_ was highly correlated (*r*^2^ = 0.97) with those calculated using genotyping arrays (*n* = 182). *x*-axis is the raw GPS calculated from the genotyping array, and *y*-axis is the raw GPS calculated from the lcWGS data; raw GPS values are unitless. lcWGS low coverage whole genome sequencing; GPS, genome-wide polygenic score; CAD, coronary artery disease; BC, breast cancer; AF, atrial fibrillation

We then calculated previously published GPSs for CAD [3], BC [10], and AF [3] on each sample using genotyping array data or lcWGS data. We found that GPS_CAD_, GPS_BC_, and GPS_AF_ were highly correlated (Fig. 3a–c), with the score mean (Student’s *t* test *p* = 0.17) and variance (*F* test *p* = 0.91) equivalent between lcWGS and the genotyping array. The correlations of GPS_CAD_ and GPS_AF_ (*r*^2^ = 0.98 and *r*^2^ = 0.97, respectively) were slightly higher than that of GPS_BC_ (*r*^2^ = 0.93). There are a few key distinctions between the GPS_BC_ and the GPS_AF_ and GPS_CAD_, which could be responsible for these differences. These include (1) the smaller number of variants in GPS_BC_ (3820 versus 6.6 million), (2) differences in allele frequencies between SNPs with high weights, and (3) GPS_BC_ which was trained and validated on a different genotyping array, the OncoArray, than the Axiom UK Biobank Array used in this study [10]. To match published scores, GPS calculation included all variants regardless of imputation quality. We observed no strong differences in the distribution of observed dosages at GPS loci between lcWGS and genotyping array (Additional file 3: Figure S3). In addition, we found no difference in average difference rates for all three GPSs between blood- and saliva-derived DNA samples using lcWGS (*p* = 0.53 for CAD, *p* = 0.21 for BC, *p* = 0.70 for AF, Additional file 3: Figure S4). We also found no differences in imputation accuracy at variants with MAF > 5% (*p* = 0.23), variants with MAF between 1 and 5% (*p* = 0.13), and variants with MAF < 1% (*p* = 0.07). This is similar to previous results that have demonstrated no differences in error profiles once coverage variability is accounted for [20].

The technical concordance cohort ranged in coverage from 0.54× to 1.76× with a mean coverage of 1.24×, and we have shown that depth can impact imputation performance—depth increases above 0.5× have a smaller but measurable effect on imputation performance (Fig. 2; Additional file 3: Figure S2). To determine the low coverage sequencing depth required for GPS accuracy, we used SAMtools [13] to downsample the lcWGS data in this cohort to 1.0×, 0.75×, 0.5×, 0.4×, 0.25×, and 0.1×. We found that GPS_CAD_, GPS_BC_, and GPS_AF_ are robust to lcWGS sequencing depth 0.5× and that coverages do not systematically bias GPS calculations in a specific direction (Additional file 3: Figures S5, S6, and S7). Interestingly, correlation at 0.1× was still high enough that GPSs at this coverage may have research utility, suggesting that significant amounts of data regarding common genetic variation could be recovered from off-target reads in exome and multi-gene panel sequencing studies to allow for GPS calculation. Taken together, these data demonstrate that lcWGS provides equivalent accuracy for calculation of GPSs, with sequencing coverage as low as 0.5×.

### Assessment of imputation performance and technical concordance across diverse populations

**Fig. 4:**
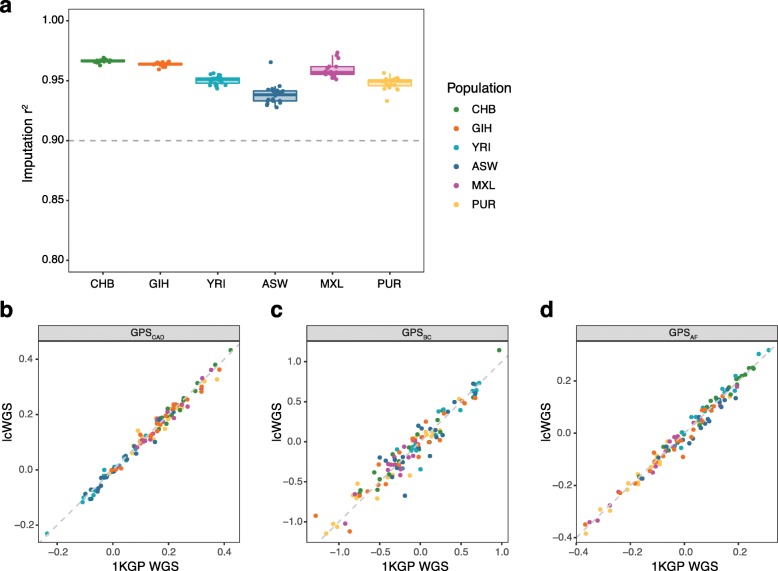
Assessment of imputation performance and technical concordance across diverse populations. **a** Imputed genotypes calculated using lcWGS data were highly correlated with genotypes from known 1KGP data (*n* = 116), with all samples having an imputation quality above 0.90 r^2^. The thin gray dashed line demonstrates previously reported imputation quality from a genotyping array (r^2^ = 0.90) [4]. **b** GPS_CAD_ calculated using lcWGS data was highly correlated (*r*^2^ = 0.98) with those calculated using known 1KGP data (*n* = 116). **c** GPS_BC_ calculated using lcWGS data was highly correlated (*r*^2^ = 0.91) with those calculated using known 1KGP data (*n* = 116). **d** GPS_AF_ was highly correlated (*r*^2^ = 0.98) with those calculated using known 1KGP data (*n* = 116). 1KGP, 1000 Genomes Project; lcWGS, low coverage whole genome sequencing; GPS, genome-wide polygenic score; CAD, coronary artery disease; BC, breast cancer; AF, atrial fibrillation

To further assess the performance of our imputation pipeline across diverse populations, we performed lcWGS on 120 additional samples from 6 1KGP populations (CHB, GIH, YRI, ASW, MXL, and PUR; Additional file 1: Table S3) that represent the range of ancestry observed in admixed populations (diverse ancestry data set) [8]. We compared genotypes imputed using our lcWGS pipeline to known 1KGP WGS data at all 21 million imputation SNP loci and found that imputation accuracy was above 0.90 *r*^2^ for all samples (range 0.94–0.97) (Fig. 4a). In addition, we found that GPS calculated from lcWGS data and GPS calculated from the Phase 3 1KGP WGS data release have a high correlation, with an *r*^2^ value of 0.98, 0.91, and 0.98 for CAD, BC, and AF, respectively (Fig. 4b–d). These results suggest that lcWGS can enable accurate imputation and calculation of GPSs in diverse populations.

### Association of lcWGS-calculated GPSs with disease phenotypes in a clinical cohort

**Fig. 5:**
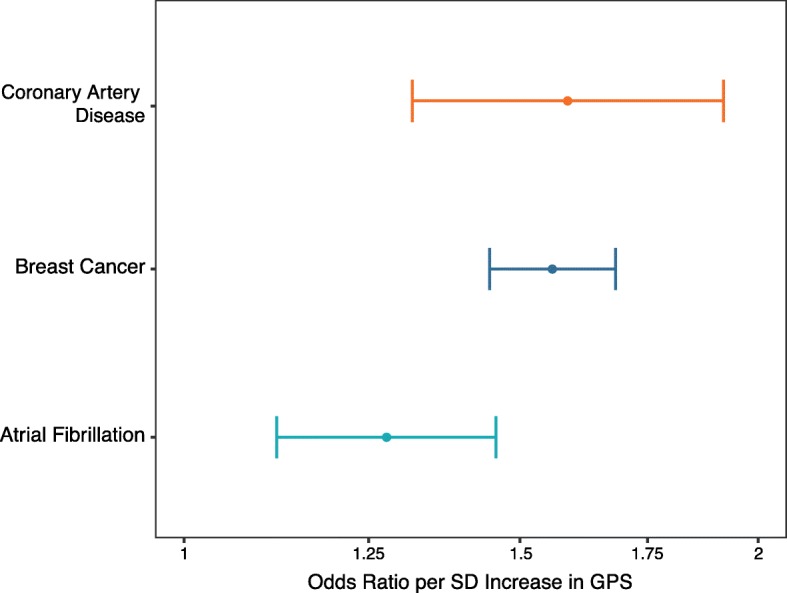
Association of lcWGS-calculated GPSs with disease phenotypes in the clinical cohort. lcWGS-calculated GPS_CAD_ was associated with personal history of CAD (OR = 1.589 (1.32–1.92), *n* = 11,010, *p* = 1.32 × 10^−6^). GPS_CAD_ was adjusted for age and sex. lcWGS-calculated GPS_BC_ was associated with personal history of BC (OR = 1.56 (1.45–1.68); *n* = 8722, *p* = 1.0 × 10^−16^). GPS_BC_ was calculated only for females and adjusted for age at menarche. lcWGS-calculated GPS_AF_ was associated with personal history of AF (OR = 1.277 (1.12–1.46); *n* = 10,303, *p* = 0.000292). GPS_AF_ was adjusted for age and sex. lcWGS, low coverage whole genome sequencing; GPS, genome-wide polygenic score; CAD, coronary artery disease; BC, breast cancer; AF, atrial fibrillation

Previous studies have demonstrated the association of GPSs with prevalent disease using genotyping arrays [3, 10, 21–23] and hcWGS [17]. To observe the performance of lcWGS-calculated GPSs in a large population, we performed low coverage sequencing on 11,502 European individuals (clinical cohort) (Additional file 1: Table S2) and calculated GPS_CAD_, GPS_BC_, and GPS_AF_ for each individual. Raw GPSs were normalized by taking the standardized residual of the predicted score after correction for the first 10 PCAs (Additional file 3: Figure S8) [17, 24]. First, we note that there are no major outliers (defined as a *z*-score greater than 5) in GPS_CAD_, GPS_BC_, and GPS_AF_ and that the normalized scores formed an approximately normal distribution for each (Additional file 3: Figure S9). Each of the GPSs was strongly associated with self-reported history of disease, with effect estimates comparable to prior reports using genotyping arrays to calculate GPS—GPS_CAD_ (OR per standard deviation = 1.59 (1.32–1.92); *n* = 11,010), GPS_BC_ (OR per standard deviation = 1.56 (1.45–1.68); *n* = 8722), and GPS_AF_ (OR per standard deviation = 1.28 (1.12–1.46); *n* = 10,303) (Fig. 5).

Area under the curve (AUC) is an additional metric used to assess the ability of a given risk factor to discriminate between affected cases and disease-free controls. When only the GPS was included in the prediction model, GPS_CAD_ had an AUC of 0.60, GPS_BC_ had an AUC of 0.63, and GPS_AF_ had an AUC of 0.57. The additional inclusion of age and sex increased the AUCs to 0.86 for GPS_CAD_, 0.78 for GPS_BC_, and 0.78 for GPS_AF_. For each of these three diseases, the magnitude of associations with clinical disease and AUC metrics was consistent with previous publications [3, 10]. Taken together, these results suggest that lcWGS-calculated GPSs can accurately stratify risk with comparable accuracy to previously published GPS-disease associations calculated on the basis of genotyping array data.

## Discussion

For the past two decades, genotyping array-based GWAS and imputation have been the driving force in our discovery of genetic loci predictive of disease and derivation and calculation of GPSs. In this study, we developed and validated an imputation pipeline to calculate GPSs from variably downsampled hcWGS and lcWGS data sets. While the efficiency of lcWGS has been reported for other applications of statistical genetics [4–6], we demonstrate that lcWGS achieves similar technical concordance as the Axiom UK Biobank Array by Affymetrix for determining GPSs. Furthermore, the imputation *r*^2^ from lcWGS was greater than 90%, which is similar to the imputation accuracy reported from other commercially available genotyping arrays [25]. Taken together, these data suggest that lcWGS has comparable accuracy to genotyping arrays for assessment of common variants and subsequent calculation of GPSs.

Our finding that lcWGS can be used for accurate genotyping and imputation of common genetic variants has implications for the future of genomic research and medicine. Currently, disease GWAS are performed using a variety of genotyping arrays designed to target specific sets of genes or features, reducing imputation quality in regions that are not targeted [26]. lcWGS enables less biased imputation than genotyping arrays by not pre-specifying the genetic content that is included for assessment, as is necessary for genotyping arrays. Because initial GWAS focused on populations with high homogeneity to reduce noise and increase fit of risk stratification, many genotyping arrays were designed to capture common genetic variants based on the linkage disequilibrium structure in European populations [27]. However, this ascertainment bias reduces the imputation performance from genotyping array data in diverse populations [28–30]. Imputation from lcWGS data reduces this bias by including all SNPs observed in 1KGP populations as potential predictors. The effects of SNP selection bias are also not equivalent across genotyping arrays, and therefore, variants included in a GPS trained and validated on one genotyping array may not be as predictive on another genotyping array [31]. lcWGS systematically surveys variants independent of SNP selection bias and thus provides one approach to overcome this issue. Our findings here demonstrate that GPSs trained and validated on different genotyping arrays are transferable to lcWGS-calculated GPS. Furthermore, as new genetic associations are discovered, lcWGS can be re-analyzed with ever more inclusive sets of identified. By contrast, genotyping arrays are static and cannot be easily updated or changed without designing a de novo platform.

lcWGS also has the potential to easily integrate into current clinical sequencing pipelines. In contrast to genotyping arrays, which require investment in separate laboratory technology, lcWGS can be performed on the same platform as current hcWGS or targeted multi-gene panel clinical testing. The ease of combining these two pathways could help to drive GPS adoption into clinical practice and can likely be achieved at a cost comparable to genotyping arrays [4]. In addition, lcWGS could be used to detect large insertions and deletions.

This study should be interpreted in the context of potential limitations. First, the imputation accuracy observed in our analysis may have been limited by the reference panel size. Future efforts using an even larger reference or more diverse panel may lead to further improved imputation accuracy, particularly for variants with allele frequency less than 1% [25, 32]. Second, while lcWGS may ultimately enable derivation of GPSs with improved predictive accuracy or ethnic transferability, this was not explicitly explored here. Rather, we demonstrate the feasibility and accuracy of using lcWGS of calculating GPSs published in previous studies. Third, disease phenotypes in our clinical cohort were based on individual self-report rather than review of health records. However, several studies have shown that self-reported personal history data have high concordance with data reported by a healthcare provider or electronic health records [33–36], and any inaccuracies would be expected to bias GPS-disease associations to the null. Fourth, while lcWGS data provides accurate inference of common variants, imputation is less accurate for rare variants. High coverage clinical sequencing of genes, such as those in the American College of Medical Genetics and Genomics (ACMG) list of genes in which pathogenic variants are deemed important and actionable [33], is essential for accurate detection of rare pathogenic variants.

## Conclusions

In conclusion, this work establishes lcWGS as an alternative approach to genotyping arrays for common genetic variant assessment and GPS calculation—providing comparable accuracy at similar cost while also overcoming the ascertainment bias inherent to variant selection in genotyping array design.

## Supplementary information

**Additional file 1: Table S1.** Samples in the pipeline validation data set. **Table S2.** Demographics of technical concordance cohort and clinical cohort. **Table S3.** Samples in the diverse ancestry data set.

**Additional file 2.** Supplementary Methods.

**Additional file 3: Figure S1.** Association of lcWGS time and accuracy for samples in the pipeline validation data set at 1.0X coverage. **Figure S2.** Imputation performance of the pipeline compared to genotyping array for different allele frequencies. **Figure S3.** Observed genotype dosages at GPS loci between lcWGS data and genotyping array. **Figure S4.** Comparison of blood-derived and saliva-derived samples. **Figure S5.** Correlation of GPSs between genotyping array and lcWGS at different coverage depths in the technical concordance cohort., **Figure S6.** Correlation of GPS_CAD_ between genotyping array and lcWGS at different coverage depths in the technical concordance cohort when removing individuals who were suspected to have a high GPS_CAD_. **Figure S7.** Concordance of GPS calculated at different coverages using different sampling seeds in the technical concordance cohort. **Figure S8.** First two principal components of ancestry. **Figure S9.** Distribution of GPSs in the clinical cohort.

## Data Availability

The technical concordance and clinical cohort data are not publicly available because the research participant consent, privacy policy, and terms of service of the commercial laboratory do not include authorization to share data. 1KGP [8], http://www.internationalgenome.org/ GIAB [9], ftp://ftp-trace.ncbi.nlm.nih.gov/giab/ftp/release/AshkenazimTrio/ Samtools/Bcftools [13], http://www.htslib.org/ BEAGLE [16], https://faculty.washington.edu/browning/beagle/beagle.html FastNGSAdmix [11], http://www.popgen.dk/software/index.php/FastNGSadmix

## Abbreviations

GPS: Genome-wide polygenic score
lcWGS: Low coverage whole genome sequencing
CAD: Coronary artery disease
BC: Breast cancer
AF: Atrial fibrillation
1KGP: 1000 Genomes Project
GIAB: Genome in a Bottle
Indel: Insertion-deletion
BWA: Burrows-Wheeler Aligner
IBS: Identity-by-State
PCs: Principal components
PCA: PC analysis
MAF: Minor allele frequency
AUC: Area under the curve
