# Supplementary material for: Low coverage whole genome sequencing enables accurate assessment of common variants and calculation of genome-wide polygenic scores
Source: Genome Med. 2019 Nov 26;11:74. doi: 10.1186/s13073-019-0682-2 (PMC6880438; doi:10.1186/s13073-019-0682-2)
Supplement: Supplementary file 1 — Additional file 1: Table S1. Samples in the pipeline validation data set. Table S2. Demographics of technical concordance cohort and clinical cohort. Table S3. Samples in the diverse ancestry data set. [file 13073_2019_682_MOESM1_ESM.pdf]

ADDITIONAL FILE 1

Supplementary tables and legends

Table S1: Samples in the pipeline validation data set.

Sample	Population code	Population description	Super population code
NA24385	AJ	Ashkenazi Jewish Trio, Son	AJ
NA24143	AJ	Ashkenazi Jewish Trio, Mother	AJ
NA24149	AJ	Ashkenazi Jewish Trio, Father	AJ
HG02155	CDX	Chinese Dai in Xishuangbanna, China	EAS
NA12878	CEU	Utah Residents with Northern and Western European Ancestry	EUR
HG00663	CHB	Southern Han Chinese	EAS
HG01485	CLM	Colombians from Medellin, Colombia	AMR
NA21144	GIH	Gujarati Indian from Houston, Texas	SAS
NA20510	TSI	Toscani in Italia	EUR
NA19420	YRI	Yoruba in Ibadan, Nigeria	AFR

AJ, Ashkenazi Jewish. EAS, East Asian. EUR, European. AMR, Ad Mixed American. SAS, South Asian. AFR, African.

Table S2: Demographics of technical concordance cohort and clinical cohort.

		Technical concordance cohort		Clinical cohort	
		Individuals (n)	Population	Individuals (n)	Population
Total		182*	100%	11,502	100%
Gender	Female	127	69.8%	9529	82.8%
	Male	55	30.2%	1973	17.1%
Age (Years)	18-30	6	3.3%	1041	9.1%
	31-40	16	8.8%	2410	21.0%
	41-50	37	20.3%	2633	22.9%
	51-65	65	35.7%	3907	34.0%
	65+	58	31.9%	1511	13.1%
Personal History	CAD	61	33.5%	126	1.1%
	BC	18†	14.1%	828†	8.6%
	AF	0	0.0%	239	2.1%

*Excludes two individuals who failed genotyping and low coverage whole genome sequencing.

†Females only. CAD, coronary artery disease. BC, breast cancer. AF, atrial fibrillation.

Table S3: Samples in the diverse ancestry data set.

Population code	Population description	Super population code	Samples			
CHB	Han Chinese in Beijing, China	EAS	NA18642<br>NA18757<br>NA18564<br>NA18609<br>NA18597	NA18619<br>NA18563<br>NA18749<br>NA18621<br>NA18534	NA18544<br>NA18555<br>NA18645<br>NA18560<br>NA18577	NA18631<br>NA18634<br>NA18543<br>NA18582<br>NA18626
YRI	Yoruba in Ibadan, Nigeria	AFR	NA18501<br>NA18502<br>NA18505<br>NA18507<br>NA18508	NA18516<br>NA18519<br>NA18861<br>NA18867<br>NA18868	NA18873<br>NA18910<br>NA18917<br>NA19095<br>NA19114	NA19117<br>NA19129<br>NA19137<br>NA19143<br>NA19147
GIH	Gujarati Indian from Houston, Texas	SAS	NA20870<br>NA20910<br>NA20886<br>NA20900<br>NA20845	NA20903<br>NA21142<br>NA21141<br>NA21105<br>NA21088	NA20899<br>NA21133<br>NA21137<br>NA21098<br>NA21114	NA21115<br>NA21103<br>NA21125<br>NA21126<br>NA21094
ASW	Americans of African Ancestry in Southwest USA	AFR	NA20314<br>NA19921<br>NA19625<br>NA19914<br>NA19818	NA19834<br>NA20359<br>NA19700<br>NA19701<br>NA20348	NA20356<br>NA19908<br>NA19916<br>NA20127<br>NA20291	NA19707<br>NA20342<br>NA20317<br>NA20351<br>NA19819
MXL	Mexican Ancestry from Los Angeles, USA	AMR	NA19729<br>NA19728<br>NA19731<br>NA19732<br>NA19678	NA19679<br>NA19651<br>NA19676<br>NA19794<br>NA19725	NA19770<br>NA19776<br>NA19661<br>NA19723<br>NA19771	NA19761<br>NA19786<br>NA19762<br>NA19785<br>NA19759
PUR	Puerto Ricans from Puerto Rico	AMR	HG01241<br>HG01188<br>HG01051<br>HG01167<br>HG01049	HG01171<br>HG01101<br>HG00734<br>HG01182<br>HG01183	HG00740<br>HG01048<br>HG01204<br>HG01098<br>HG01248	HG00551<br>HG01067<br>HG01104<br>HG01197<br>HG00640

EAS, East Asian. AFR, African. SAS, South Asian. AMR, Ad Mixed American.
