# Supplementary material for: Low coverage whole genome sequencing enables accurate assessment of common variants and calculation of genome-wide polygenic scores
Source: Genome Med. 2019 Nov 26;11:74. doi: 10.1186/s13073-019-0682-2 (PMC6880438; doi:10.1186/s13073-019-0682-2)
Supplement: Supplementary file 2 — Additional file 2. Supplementary Methods. [file 13073_2019_682_MOESM2_ESM.pdf]

ADDITIONAL FILE 2

Supplementary Methods

The multi-gene NGS panel test analyzed genes that have been associated with an elevated risk of hereditary cancer or hereditary heart conditions. These genes were selected based on published evidence of association and technical feasibility using the methods described. Analysis, variant calling, and reporting focused on the complete coding sequence and adjacent intronic sequence of the primary transcript(s), unless otherwise indicated*.

For hereditary cancer, these genes are *APC, ATM, BAP1, BARD1, BMPR1A, BRCA1, BRCA2, BRIP1, CDH1, CDK4*, CDKN2A* (p14ARF and p16INK4a), *CHEK2, EPCAM*, GREM1*, MITF*, MLH1, MSH2, MSH6, MUTYH, NBN, PALB2, PMS2*, POLD1*, POLE*, PTEN, RAD51C, RAD51D, SMAD4, STK11,* and *TP53*. Exons 12-15 of *PMS2* cannot be reliably assessed with standard target enrichment protocols. For the *CDK4*, *MITF*, *POLD1,* and *POLE*, the elevated risk of cancer is associated with distinct functional genomic regions; therefore, the complete coding sequences of these genes were not reported, but instead only the following regions: *CDK4* - chr12:g.58145429-58145431 (codon 24), *MITF* - chr3:g.70014091 (including c.952G>A), *POLD1* - chr19:g.50909713 (including c.1433G>A) and *POLE* - chr12:g.133250250 (including c.1270C>G). For *EPCAM*, only deletions including the 3' end of the gene (exons 8 and/or 9) were reported. *GREM1* was only analyzed for duplications in the upstream regulatory region.

For hereditary heart conditions, these genes are *ACTA2*, *ACTC1*, *APOB**, *COL3A1*, *DSC2*, *DSG2*, *DSP*, *FBN1*, *GLA*, *KCNH2**, *KCNQ1**, *LDLR**, *LMNA*, *MYBPC3*, *MYH7**, *MYH11*, *MYL2*, *MYL3*, *PCSK9*, *PKP2*, *PRKAG2*, *RYR2*, *SCN5A*, *SMAD3*, *TGFBR1**, *TGFBR2*, *TMEM43*,

TNNI3, *TNNT2*, and *TPM1*. *APOB* exon 1, *KCNH2* exon 4, *KCNQ1* exon 1 and *TGFBR1* exon 1 cannot be reliability assessed with standard target enrichment protocols. For the *LDLR* promoter region, the detection of deletions, duplications, and complex structural rearrangements may be limited. For the *LDLR* promoter region, the detection of deletions, duplications, and complex structural rearrangements may be limited.

Variants were classified according to the American College of Medical Genetics and Genomics 2015 guidelines for sequence variant interpretation, and all variant classifications were signed out by a board certified medical geneticist or pathologist.
