# Supplementary material for: Low coverage whole genome sequencing enables accurate assessment of common variants and calculation of genome-wide polygenic scores
Source: Genome Med. 2019 Nov 26;11:74. doi: 10.1186/s13073-019-0682-2 (PMC6880438; doi:10.1186/s13073-019-0682-2)
Supplement: Supplementary file 3 — Additional file 3: Figure S1. Association of lcWGS time and accuracy for samples in the pipeline validation data set at 1.0X coverage. Figure S2. Imputation performance of the pipeline compared to genotyping array for different allele frequencies. Figure S3. Observed genotype dosages at GPS loci between lcWGS data and genotyping array. Figure S4. Comparison of blood-derived and saliva-derived samples. Figure S5. Correlation of GPSs between genotyping array and lcWGS at different coverage depths in the technical concordance cohort., Figure S6. Correlation of GPSCAD between genotyping array and lcWGS at different coverage depths in the technical concordance cohort when removing individuals who were suspected to have a high GPSCAD. Figure S7. Concordance of GPS calculated at different coverages using different sampling seeds in the technical concordance cohort. Figure S8. First two principal components of ancestry. Figure S9. Distribution of GPSs in the clinical cohort. [file 13073_2019_682_MOESM3_ESM.pdf]

# Figure S1

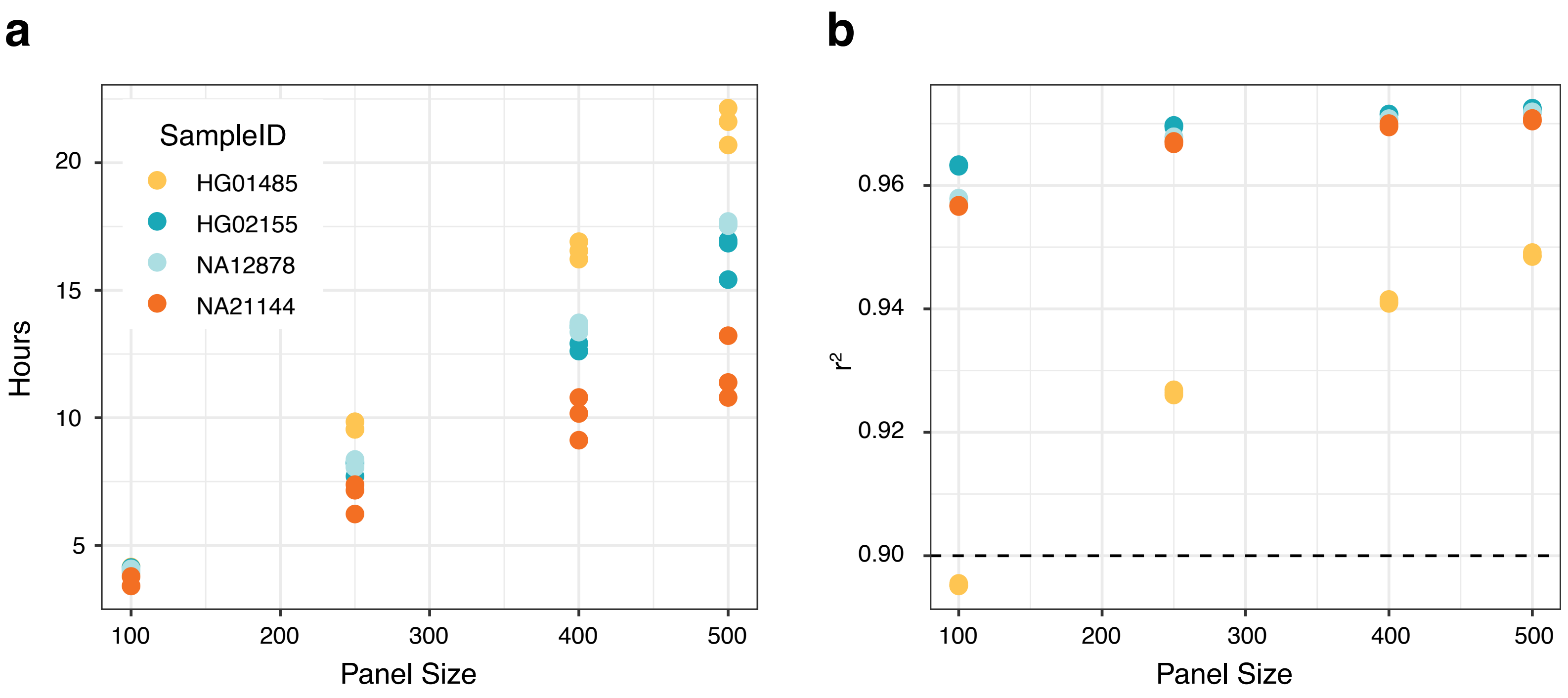


Figure S1. Association of lcWGS time and accuracy for samples in the pipeline validation data set at 1.0X coverage. (A) Imputation time increased as the size of the reference panel increased. (B) Imputation accuracy increased as the size of the reference panel increased, with less improvement after a panel size of 250. lcWGS, low coverage whole genome sequencing.

# Figure S2

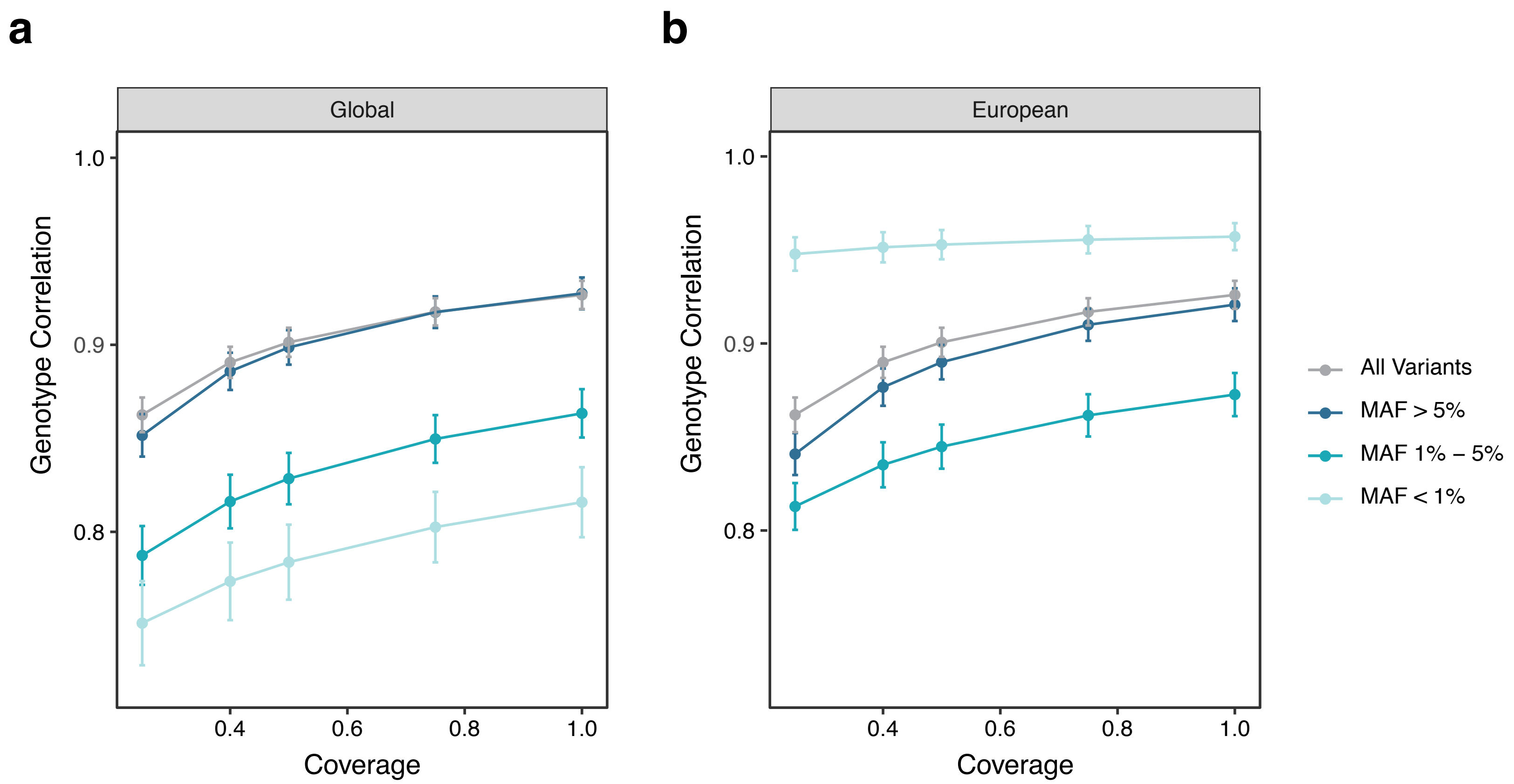


Figure S2. Imputation performance of the pipeline compared to genotyping array for different allele frequencies. (A) Imputation quality was highest for variants above 5% global MAF but was reduced for variants with lower allele frequencies. (B) Imputation quality for variants below 0.1% European MAF was inflated due to the high number of reference variants. MAF, minor allele frequency.

# Figure S3

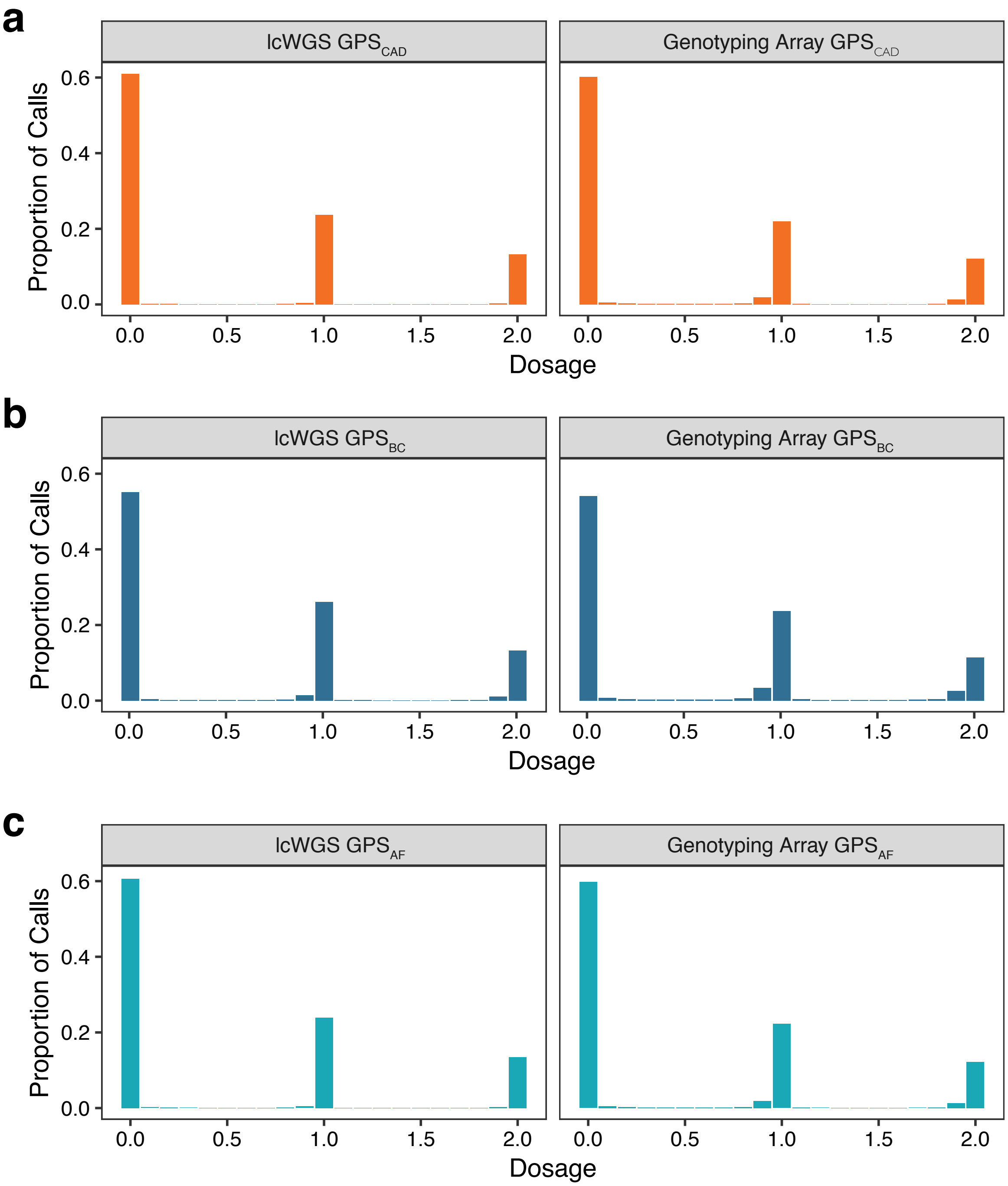


Figure S3. Observed genotype dosages at GPS loci between lcWGS data and genotyping array. (A) Dosage distributions across all imputed variants were similar for GPS$_{CAD}$ calculated from lcWGS data and genotyping array. (B) Dosage distributions across all imputed variants were similar for GPS$_{BC}$ calculated from lcWGS data and genotyping array. (C) Dosage distributions across all imputed variants were similar for GPS$_{AF}$ calculated from lcWGS data and genotyping array. lcWGS, low coverage whole genome sequencing. CAD, coronary artery disease. BC, breast cancer. AF, atrial fibrillation.

# Figure S4

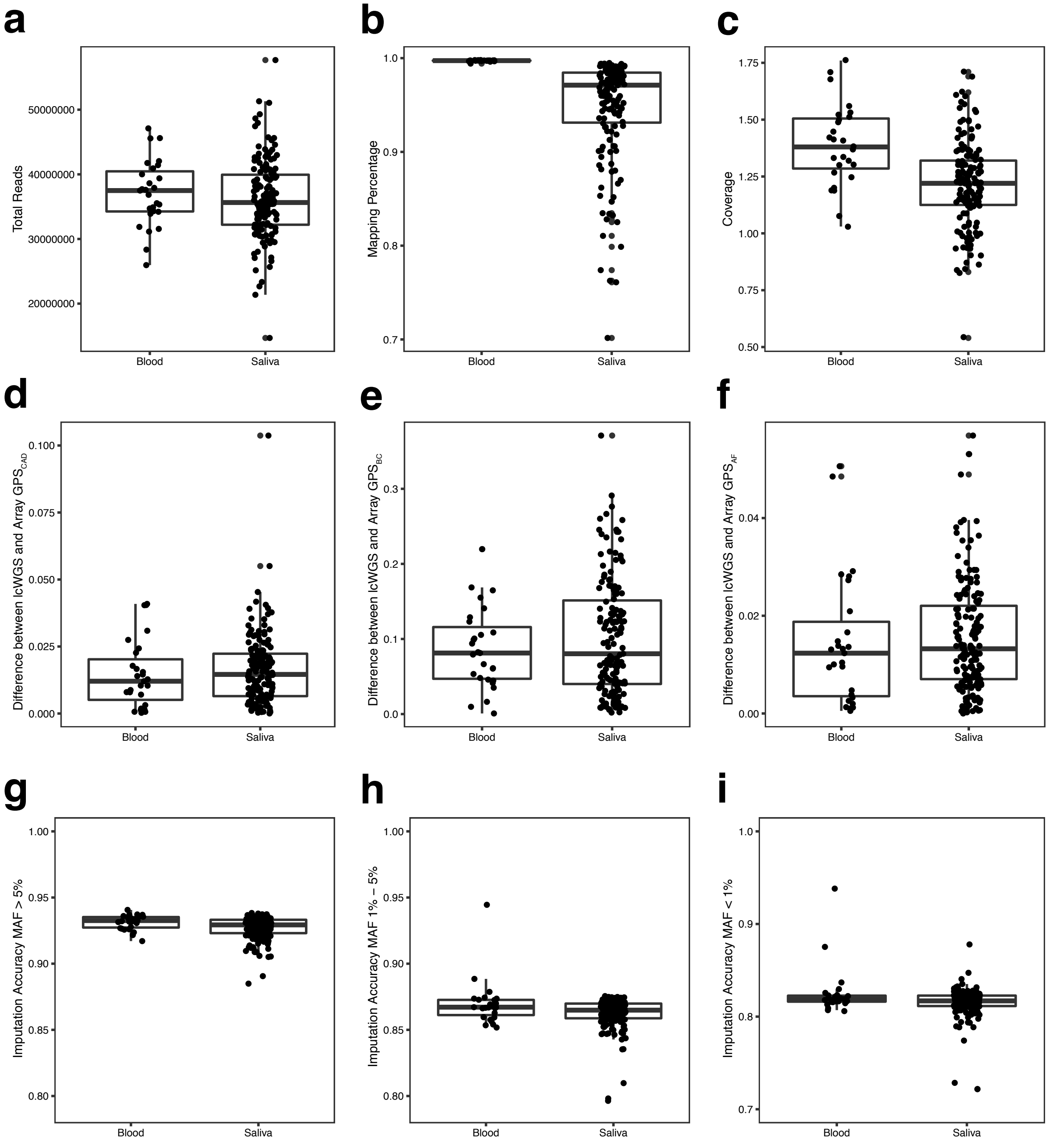


Figure S4. Comparison of blood-derived and saliva-derived samples. (A) Total reads were similar in blood-derived and saliva-derived samples ($p = 0.51$). (B) Mapping percentage was higher in blood-derived samples than saliva-derived samples ($p < 2 \times 10^{-16}$). (C) Overall coverage was higher in blood-derived samples than saliva-derived samples ($p = 6.0 \times 10$-4). (D) $GPS_{CAD}$ was similar in blood-derived samples and saliva-derived samples ($p = 0.50$). (E) $GPS_{BC}$ was similar in blood-derived samples and saliva-derived samples ($p = 0.2$). (F) $GPS_{AF}$ was similar in blood-derived samples and saliva-derived samples ($p = 0.7$). lcWGS, low coverage whole genome sequencing. GPS, genome-wide polygenic score. CAD, coronary artery disease. BC, breast cancer. AF, atrial fibrillation. (G) Imputation accuracy at variants with MAF > 5% was similar between blood-derived and saliva-derived samples once coverage was accounted for ($p = 0.23$). (H) Imputation accuracy at variants with MAF between 1% and 5% was similar between blood-derived and saliva-derived samples once coverage was accounted for ($p = 0.13$). (I) Imputation accuracy at variants with MAF < 1% was similar between blood-derived and saliva-derived samples once coverage was accounted for ($p = 0.07$).

# Figure S5

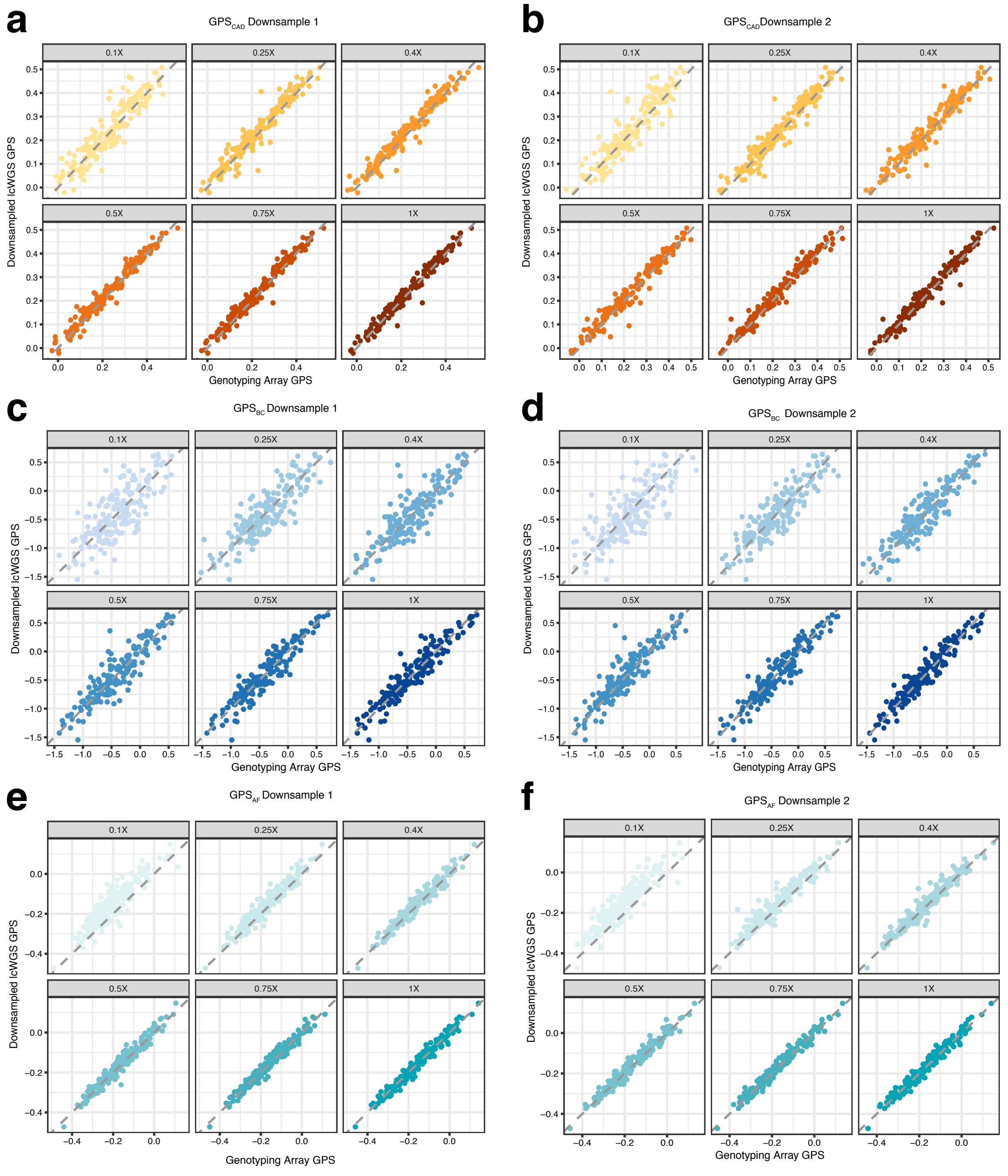


Figure S5. Correlation of GPSs between genotyping array and lcWGS at different coverage depths in the technical concordance cohort. (A, B) Downsampling from 1.0X to 0.1X showed that GPSCAD calculated using lcWGS was highly correlated with the genotyping array at 1.0X to 0.5X coverage but decreased at 0.1X (n = 182, two independent random seeds). (C, D) Downsampling from 1.0X to 0.1X showed that GPSBC calculated using lcWGS was highly correlated with the genotyping array at 1.0X to 0.5X coverage but decreased at 0.1X (n = 182, two independent random seeds). (D, E) Downsampling from 1.0X to 0.1X showed that GPSAF calculated using lcWGS was highly correlated with the genotyping array at 1.0X to 0.5X coverage but decreased at 0.1X (n = 182, two independent random seeds). x-axis is the raw GPS calculated from the genotyping array, and y-axis is the raw GPS calculated from the lcWGS data; raw GPS values are unitless. lcWGS, low coverage whole genome sequencing. GPS, genome-wide polygenic score. CAD, coronary artery disease. BC, breast cancer. AF, atrial fibrillation.

# Figure S6

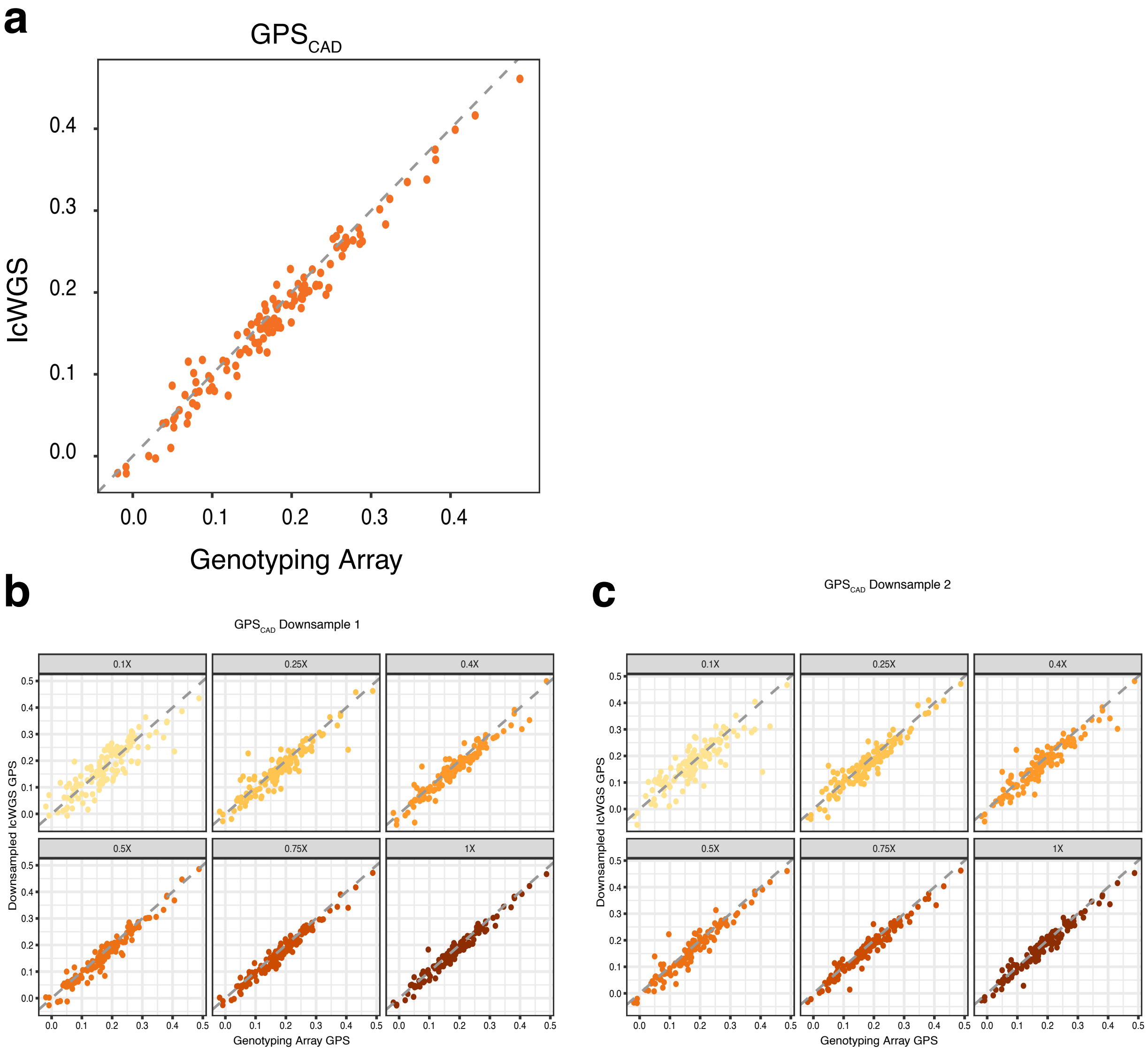


Figure S6. Correlation of $GPS_{CAD}$ between genotyping array and lcWGS at different coverage depths in the technical concordance cohort when removing individuals who were suspected to have a high $GPS_{CAD}$. (A) The analyzed technical concordance cohort included 59 individuals who were suspected to have a high $GPS_{CAD}$ based on targeted panel and off target sequencing data, which could artificially inflate the observed correlation above what would be seen in a randomly selected sample. $GPS_{CAD}$ remained highly correlated after removing those individuals who were suspected to have a high $GPS_{CAD}$ based on targeted panel and off target sequencing data ($r^2 = 0.97$, n = 123). (B, C) Downsampling from 1.0X to 0.1X showed that $GPS_{CAD}$ calculated using lcWGS was highly correlated with the genotyping array at 1.0X to 0.5X coverage but decreased at 0.1X (n = 123, two independent random seeds). x-axis is the raw GPS calculated from the genotyping array, and y-axis is the raw GPS calculated from the lcWGS data; raw GPS values are unitless. lcWGS, low coverage whole genome sequencing. GPS, genome-wide polygenic score. CAD, coronary artery disease.

# Figure S7

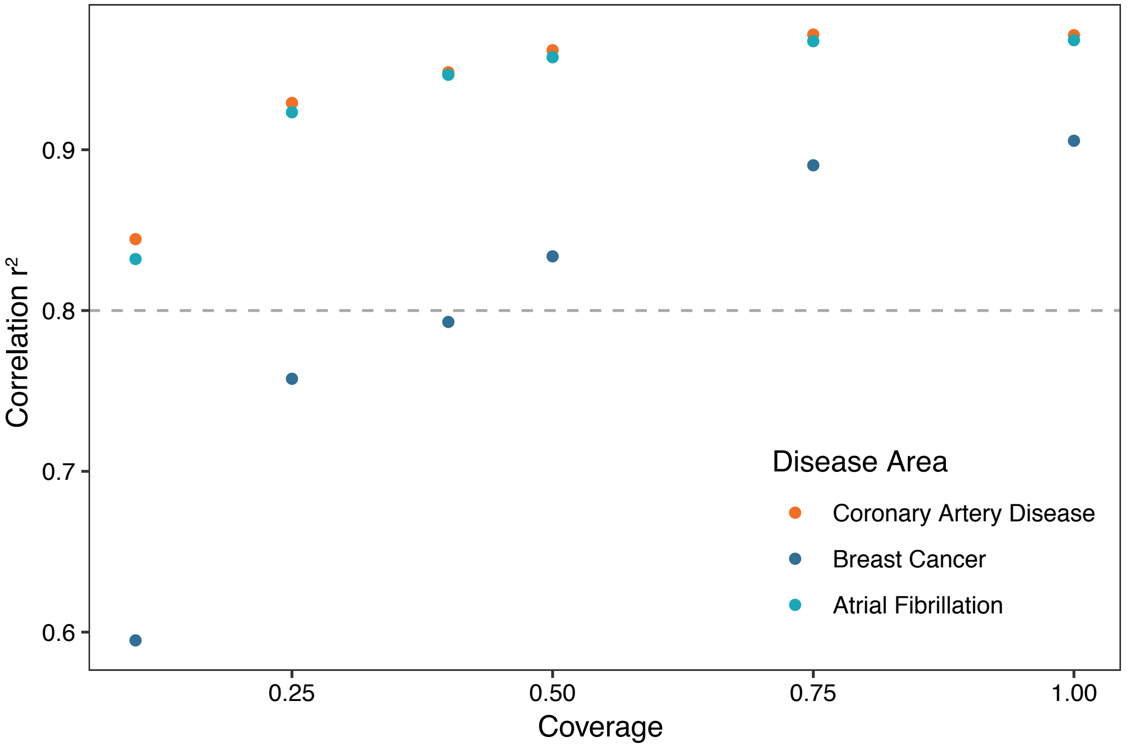


Figure S7. Concordance of GPS calculated at different coverages using different sampling seeds in the technical concordance cohort. GPS concordance is > 0.80 $r^2$ when samples were sequenced at or above 0.5X coverage (n = 182) ($GPS_{CAD}$ p = 0.004, $GPS_{BC}$ p = 0.0001, $GPS_{AF}$ p = 0.004). GPS, genome-wide polygenic score. CAD, coronary artery disease. BC, breast cancer. AF, atrial fibrillation.

# Figure S8

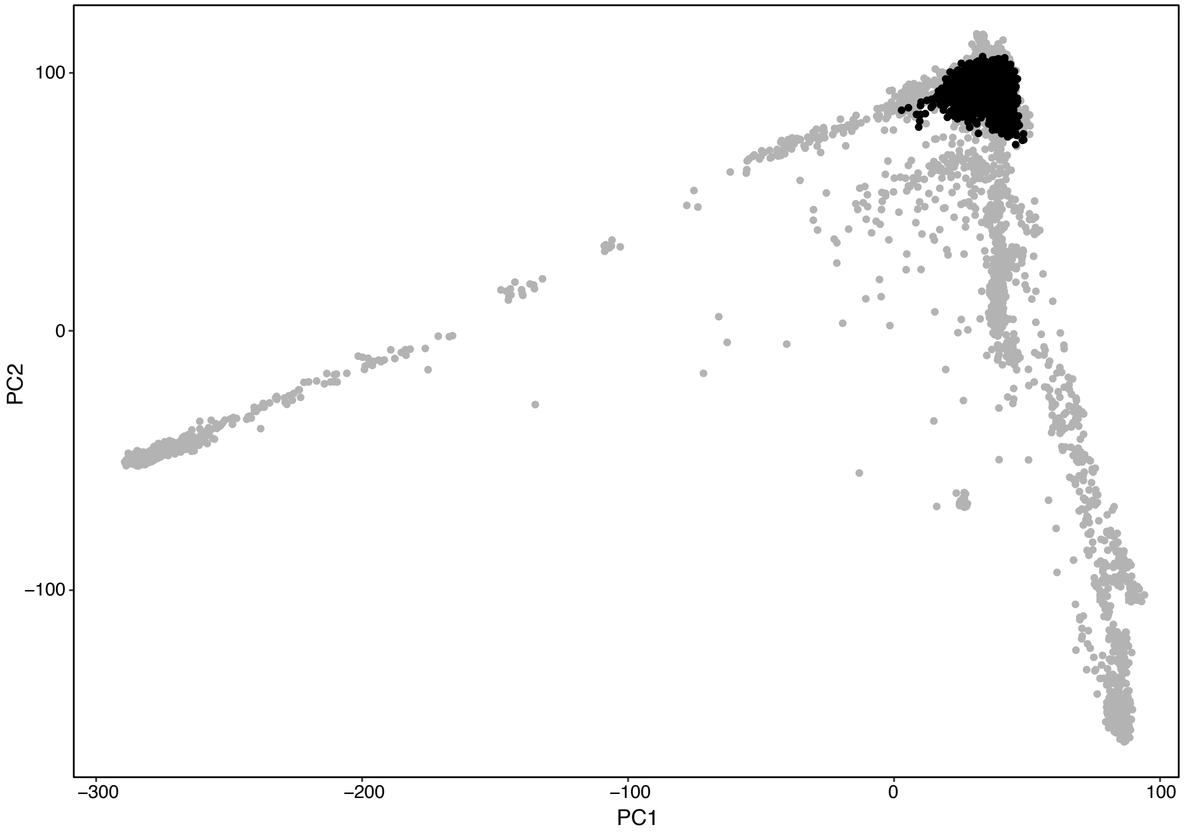


Figure S8. First two principal components of ancestry. Raw GPSs were normalized by taking the standardized residual of the predicted score after correction for the first 10 principal components (PC) of ancestry. Grey points correspond to reference samples from the 1KGP and Human Origins. Black points represent individuals in the clinical cohort in this study. GPS, genome-wide polygenic score. 1KGP, 1000 Genome Project.

# Figure S9

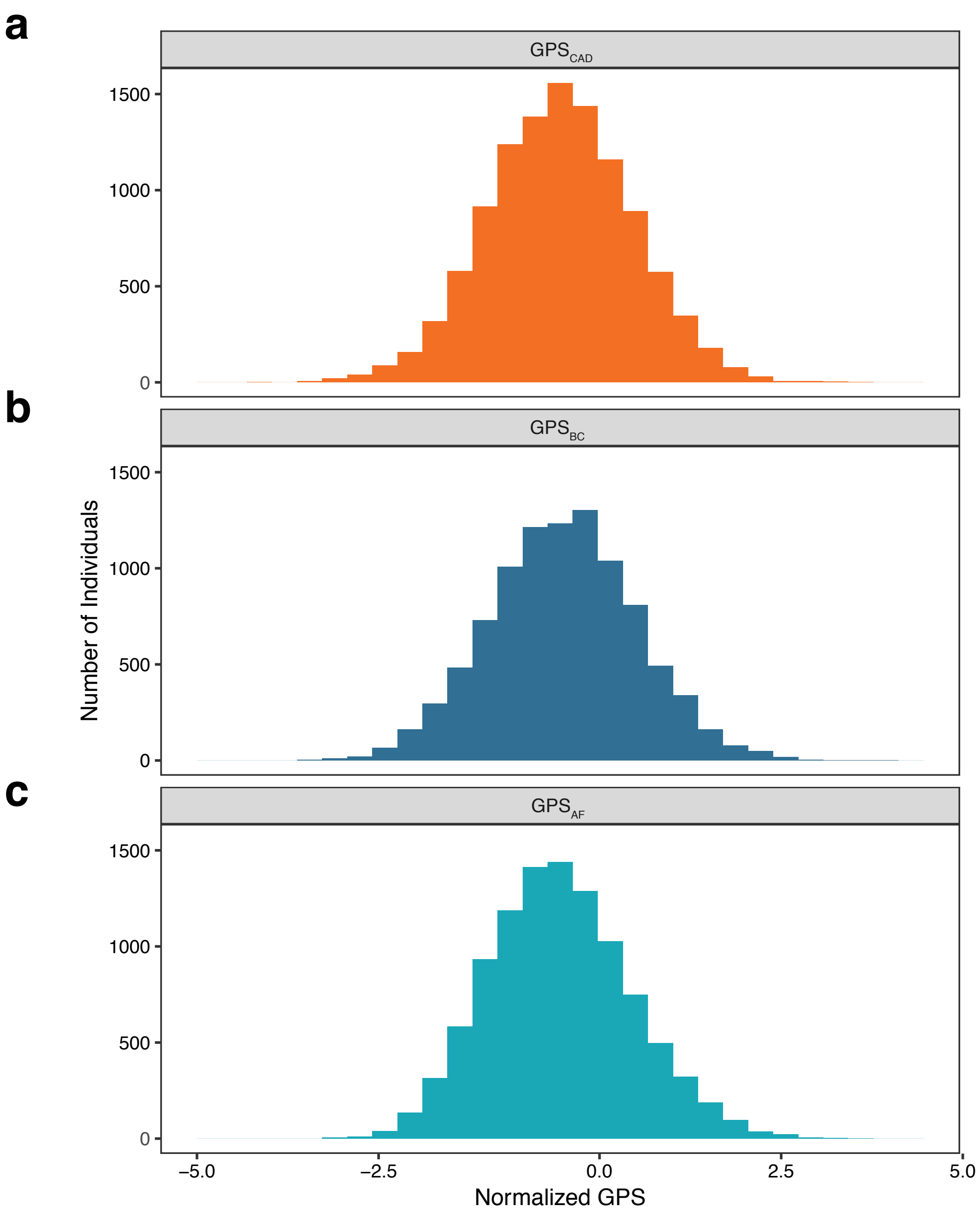


Figure S9. Distribution of GPSs in the clinical cohort. (A) The distribution of $GPS_{CAD}$ was approximately normal in 11,010 individuals (p = 1.32 x $10^{-6}$). (B) The distribution of $GPS_{BC}$ was approximately normal in 8,722 individuals ( p = 1.0 x $10^{-16}$). (C) The distribution of $GPS_{AF}$ was approximately normal in 10,303 individuals (p = 0.000292). GPS, genome-wide polygenic score. CAD, coronary artery disease. BC, breast cancer. AF, atrial fibrillation.
